# Editorial: Sexual health week 2022

**DOI:** 10.3389/frph.2024.1479667

**Published:** 2024-11-15

**Authors:** Abdulbasit Seid

**Affiliations:** School of Public Health and Preventive Medicine, Monash University, Melbourne, VIC, Australia

**Keywords:** sexual reproductive health services, pre exposure prophylaxis (PrEP), sexually tranmistted infections, adolescent, access and utilisation

**Editorial on the research topic**
Sexual health week 2022

The World Health Organization (WHO) describes sexual health as “a state of physical, emotional, mental, and social well-being related to sexuality, not just the absence of disease, dysfunction, or infirmity” (1). Ensuring universal access to Sexual and Reproductive Health Services (SRHS) is essential for achieving Sustainable Development Goals 3 and 5 (2). Over the past three decades, remarkable progress in improving access to and utilization of SRHS has significantly enhanced the lives of millions. However, this progress has recently stagnated and, in some countries, is even backsliding (3).

Despite the WHO's global strategies to improve SRHS by focusing on key areas such as improving family planning services, eliminating unsafe abortion, combatting sexually transmitted infections (STIs), and promoting sexual health, the disparities among countries persist (4). Several factors contribute to these disparities, including but not limited to varying levels of political commitment to ensuring SRHR (5), cultural influences (6), racial and ethnicity differences (7, 8), and provider-related factors (9).

This Special Issue covers several aspects of sexual and reproductive health, focusing on (1) adolescents’ and young people's access to and use of SRHS and (2) STI and strategies to improve the pre-exposure prophylaxis (PrEP) for HIV prevention.

## Adolescents’ and young people's access to and use of SRHS

Adolescents and young people often face significant challenges in accessing and using SRHS (10). These challenges are diverse and vary from country to country, with major obstacles including inadequate knowledge, political and socio-cultural barriers, economic constraints, and negative attitude of healthcare providers (11). A recent study by Mejia et al. highlighted a significant gap in sexual and reproductive health knowledge among adolescents and young people, identifying a lack of information about sex, reproduction, and intimate and romantic relationships as the main contributing factors. This study underscored the critical need to address this gap by providing the necessary sexual and reproductive health education for youth.

Lack of knowledge, combined with other legal and socio-cultural barriers, puts adolescents and young people at a higher risk of sexual and reproductive health problems. For example, evidence has indicated that, in 2023, adolescents accounted for over 12% of all new Human Immunodeficiency Virus (HIV) infections, with adolescent girls representing more than two-thirds of these cases (12). Similarly, the risk of unwanted pregnancy is high among women aged 15–19 years, with an estimated 41.3 births per 1,000 women in 2023 (13). In low- and middle-income countries, the adolescent birth rate is even higher, reaching 97.9 per 1,000 women in 2023 (13). A study in this special issue by Ndagijimana et al. noted that 41% of in-school adolescents experienced risky sexual behavior in their lifetime, including not using any protection during sexual intercourse. Factors such as sexual violence, alcohol misuse, peer pressure, and lack of family support were found to increase the likelihood of engaging in risky sexual behavior. A similar study by Kawuma et al. identified important barriers to accessing broader contraceptives among sexually active adolescent girls and young women, such as age, misinformation about contraceptives, influence from partners, and family pressure.

Evidence indicates that adolescents often prefer services that address their needs with friendly care and appropriate support (14). To date, various interventions have been employed to ensure adolescents receive proper care, with varying degrees of success (15). School-based health centers, as one such intervention, offer essential services to adolescents who struggle to access regular primary care or need extra assistance in interacting with SRHS providers (16). For example, a recent study (Perez and Kelley) found that 62% of teens visited school-based health centers primarily for birth control. This study also showed that teens understood the dangers of unprotected sex and were willing to make responsible health decisions, underscoring the importance of school-based health centers in supporting adolescents’ sexual and reproductive health.

Overall, the studies included in this section of the research topic identified additional barriers that adolescents often face and emphasized the importance of providing adolescent-friendly SRHS, including school-based health centers, to ensure proper access to care.

## STIs and strategies to improve the uptake of PrEP for HIV prevention

STIs are among the leading communicable diseases, affecting millions of people in the world and with an estimated one million new cases per year (17). In an effort to tackle the spread of STIs, the WHO recommends that countries establish strong STI surveillance systems to monitor trends, identify outbreaks, redirect resources, and assess the effectiveness of interventions (18). The WHO Global Sexually Transmitted Infection Surveillance Priorities for Action identified several key priorities for countries. These priorities include strengthening and integrating STI surveillance into national health information systems, increasing the detail and specificity of data, identifying specific populations at risk, including data on risk factors and determinants of STIs, and strengthening national laboratory capacity (19). The implementation of STI surveillance varies among countries, contributing to a lack of adequate understanding of STIs globally (20). To enhance this understanding, Wang et al. conducted a study investigating the prevalence of *Ureaplasma urealyticum*, *Chlamydia trachomatis*, and *Neisseria gonorrhoeae* among at-risk populations. Their findings indicated higher rates of infections among younger populations and women, highlighting the need for regular screening to improve detection and management of these infections.

Prevention and treatment of STIs are considered cornerstone strategies in the prevention of HIV infections (21). Additionally, specific measures are available for people at risk of acquiring HIV to reduce their risk of infection (22). PrEP is one of the key measures for reducing the spread of HIV and is recommended by the WHO as part of comprehensive HIV prevention strategies (23). In improving the uptake of PrEP, various strategies such as pharmacy-based PrEp provision, covering PrEP cost through Medicaid, and offering PrEP with delivery of positive STI results have been implemented (24). However, these strategies vary across settings based on the nature of healthcare systems and available resources. To further enhance PrEP uptake, Reyniers et al. identified three key strategies to improve uptake among men who have sex with men. These strategies include raising awareness and increasing knowledge of HIV to enhance perceived risk, improving the dissemination of PrEP, and employing methods to avoid stigma and discrimination.

In conclusion, the articles included in this section of the Special Issue highlight the persistent and significant threat posed by STIs. It also provides critical strategies that need to be considered and implemented to enhance the uptake of PrEP, ultimately contributing to the reduction of HIV transmission.

## References

[1] WHO. Sexual Health, Human Rights and the Law. Geneva, Switzerland: World Health Organization (2015).

[2] United Nations. Sustainable development. (2015). Available online at: https://sdgs.un.org/goals (accessed July 22, 2024).

[3] WHO, UNFPA, UNAIDS, UNICEF, UN WOMEN. Joint UN Statement: Calling for Sexual and Reproductive Health and Rights for all. Geneva: World Health Organization (2024).

[4] WHO. Sexual and reproductive health for all: 20 years of the GLOBAL strategy. (2023). Available online at: https://www.who.int/news/item/16-05-2024-sexual-and-reproductive-health-for-all-20-years-of-the-global-strategy (accessed July 22, 2024).

[5] Pugh S. Politics, power, and sexual and reproductive health and rights: impacts and opportunities. Sex Reprod Health Matters. (2019) 27(2):1–5. 10.1080/26410397.2019.1662616 31524106 PMC7887954

[6] Barrio-Ruiz C Ruiz de Viñaspre-Hernandez R Colaceci S Juarez-Vela R Santolalla-Arnedo I Durante A Language and cultural barriers and facilitators of sexual and reproductive health care for migrant women in high-income European countries: an integrative review. J Midwifery Womens Health. (2024) 69(1):71–90. 10.1111/jmwh.13545 37531180

[7] Sutton MY Anachebe NF Lee R Skanes H. Racial and ethnic disparities in reproductive health services and outcomes, 2020. Obstet Gynecol. (2021) 137(2):225–233. 10.1097/AOG.0000000000004224 33416284 PMC7813444

[8] Rosenthal L Lobel M. Gendered racism and the sexual and reproductive health of black and Latina women. Ethn Health. (2020) 25(3):367–92. 10.1080/13557858.2018.1439896 29447448

[9] WHO. Social Determinants of Sexual and Reproductive Health. Geneva: World Health Organization (2010).

[10] WHO. Adolescent sexual and reproductive health. (2024). Available online at: https://www.who.int/southeastasia/activities/adolescent-sexual-reproductive-health (accessed July 18, 2024).

[11] Morris JL Rushwan H. Adolescent sexual and reproductive health: the global challenges. Int J Gynaecol Obstet. (2015) 131(S1):S40–2. 10.1016/j.ijgo.2015.02.006 26433504

[12] UNICEF. Adolescents HIV prevention. (2024). Available online at: https://data.unicef.org/topic/hivaids/adolescents-young-people/ (accessed July 18, 2024).

[13] WHO. Adolescent pregnancy. (2024). Available online at: https://www.who.int/news-room/fact-sheets/detail/adolescent-pregnancy (accessed July 19, 2024).

[14] World Health Organization. Strategic Directions for Improving Adolescent Health in South-East Asia Region. New Delhi: Regional Office for South-East Asia (2011).

[15] Salam RA Faqqah A Sajjad N Lassi ZS Das JK Kaufman M Improving adolescent sexual and reproductive health: a systematic review of potential interventions. J Adolesc Health. (2016) 59(4S):S11–28. 10.1016/j.jadohealth.2016.05.022 27664592 PMC5026684

[16] National Research Council and Institute of Medicine. Adolescent health services: missing opportunities. In: Gootman JA Lawrence RS Sim LJ, editors. Current Adolescent Health Services, Settings, and Providers. Washington (DC): National Academies Press (2009). 25009892

[17] Sinka K. The global burden of sexually transmitted infections. Clin Dermatol. (2024) 42(2):110–8. 10.1016/j.clindermatol.2023.12.002 38142791

[18] World Health Organization. Strategies and Laboratory Methods for Strengthening Surveillance of Sexually Transmitted Infection. Geneva: World Health Organization (2015).

[19] World Health Organization. Global health sector strategy on sexually transmitted infections, 2016–2021 (2019). Available online at: https://www.who.int/publications/i/item/WHO-RHR-16.09 (accessed July 15, 2024).

[20] Mohammed H Hughes G Fenton KA. Surveillance systems for sexually transmitted infections: a global review. Curr Opin Infect Dis. (2016) 29(1):64–9. 10.1097/QCO.0000000000000235 26658655

[21] Cohen MS Council OD Chen JS. Sexually transmitted infections and HIV in the era of antiretroviral treatment and prevention: the biologic basis for epidemiologic synergy. J Int AIDS Soc. (2019) 22(S6):e25355. 10.1002/jia2.25355 31468737 PMC6715951

[22] Kumah E Boakye DS Boateng R Agyei E. Advancing the global fight against HIV/aids: strategies, barriers, and the road to eradication. Ann Glob Health. (2023) 89(1):83. 10.5334/aogh.4277 38046536 PMC10691281

[23] World Health Organization. WHO Implementation Tool for Pre-exposure Prophylaxis (PrEP) of HIV Infection: Provider Module for Oral and Long-acting PrEP. Geneva: World Health Organization (2024).

[24] Sullivan PS Mena L Elopre L Siegler AJ. Implementation strategies to increase PrEP uptake in the south. Curr HIV/AIDS Rep. (2019) 16(4):259–69. 10.1007/s11904-019-00447-4 31177363 PMC7117066

